# Hyperaemic effects of adenosine and dobutamine in heart failure with reduced ejection fraction: a quantitative perfusion CMR study

**DOI:** 10.1093/ehjimp/qyag128

**Published:** 2026-07-16

**Authors:** Simran Shergill, Ahmed M S E K Abdelaty, Sunny R Sedani, Aida Moafi, Lavanya Athithan, Joanna M Bilak, Mohamed Elshibly, Iyare Nehikhare, Adeogo A Olusan, Pharveen Jaspal, Simon L Hetherington, Mohamed Alama, Peter Kellman, David Adlam, Eylem Levelt, Anvesha Singh, Iain B Squire, Sandeep S Hothi, Jeffrey Khoo, Gaurav S Gulsin, Gerry P McCann, J Ranjit Arnold

**Affiliations:** Division of Cardiovascular Sciences, University of Leicester, the National Institute for Health and Care Research Biomedical Research Centre Leicester and British Heart Foundation Centre of Research Excellence, Glenfield Hospital, Groby Road, Leicester LE3 9QP, UK; Division of Cardiovascular Sciences, University of Leicester, the National Institute for Health and Care Research Biomedical Research Centre Leicester and British Heart Foundation Centre of Research Excellence, Glenfield Hospital, Groby Road, Leicester LE3 9QP, UK; Division of Cardiovascular Sciences, University of Leicester, the National Institute for Health and Care Research Biomedical Research Centre Leicester and British Heart Foundation Centre of Research Excellence, Glenfield Hospital, Groby Road, Leicester LE3 9QP, UK; Division of Cardiovascular Sciences, University of Leicester, the National Institute for Health and Care Research Biomedical Research Centre Leicester and British Heart Foundation Centre of Research Excellence, Glenfield Hospital, Groby Road, Leicester LE3 9QP, UK; Division of Cardiovascular Sciences, University of Leicester, the National Institute for Health and Care Research Biomedical Research Centre Leicester and British Heart Foundation Centre of Research Excellence, Glenfield Hospital, Groby Road, Leicester LE3 9QP, UK; Division of Cardiovascular Sciences, University of Leicester, the National Institute for Health and Care Research Biomedical Research Centre Leicester and British Heart Foundation Centre of Research Excellence, Glenfield Hospital, Groby Road, Leicester LE3 9QP, UK; Division of Cardiovascular Sciences, University of Leicester, the National Institute for Health and Care Research Biomedical Research Centre Leicester and British Heart Foundation Centre of Research Excellence, Glenfield Hospital, Groby Road, Leicester LE3 9QP, UK; Department of Cardiology, University Hospitals of Leicester NHS Trust, Glenfield Hospital, Leicester, UK; Department of Cardiology, University Hospitals of Leicester NHS Trust, Glenfield Hospital, Leicester, UK; Department of Medicine, South Warwickshire University NHS Foundation Trust, Warwick, UK; Department of Cardiology, Kettering General Hospital, Kettering, UK; Department of Cardiology, Kettering General Hospital, Kettering, UK; Department of Health and Human Services, National Heart, Lung, and Blood Institute, National Institutes of Health, Bethesda, MD, USA; Division of Cardiovascular Sciences, University of Leicester, the National Institute for Health and Care Research Biomedical Research Centre Leicester and British Heart Foundation Centre of Research Excellence, Glenfield Hospital, Groby Road, Leicester LE3 9QP, UK; Department of Cardiometabolic Imaging, Baker Heart and Diabetes Institute, Melbourne, Australia; University of Melbourne, Melbourne, Australia; Division of Cardiovascular Sciences, University of Leicester, the National Institute for Health and Care Research Biomedical Research Centre Leicester and British Heart Foundation Centre of Research Excellence, Glenfield Hospital, Groby Road, Leicester LE3 9QP, UK; Division of Cardiovascular Sciences, University of Leicester, the National Institute for Health and Care Research Biomedical Research Centre Leicester and British Heart Foundation Centre of Research Excellence, Glenfield Hospital, Groby Road, Leicester LE3 9QP, UK; Heart and Lung Centre, Royal Wolverhampton NHS Trust, Wolverhampton, UK; Institute of Cardiovascular Sciences, University of Birmingham, Birmingham, UK; Division of Cardiovascular Sciences, University of Leicester, the National Institute for Health and Care Research Biomedical Research Centre Leicester and British Heart Foundation Centre of Research Excellence, Glenfield Hospital, Groby Road, Leicester LE3 9QP, UK; Department of Cardiology, University Hospitals of Leicester NHS Trust, Glenfield Hospital, Leicester, UK; Division of Cardiovascular Sciences, University of Leicester, the National Institute for Health and Care Research Biomedical Research Centre Leicester and British Heart Foundation Centre of Research Excellence, Glenfield Hospital, Groby Road, Leicester LE3 9QP, UK; Division of Cardiovascular Sciences, University of Leicester, the National Institute for Health and Care Research Biomedical Research Centre Leicester and British Heart Foundation Centre of Research Excellence, Glenfield Hospital, Groby Road, Leicester LE3 9QP, UK; Division of Cardiovascular Sciences, University of Leicester, the National Institute for Health and Care Research Biomedical Research Centre Leicester and British Heart Foundation Centre of Research Excellence, Glenfield Hospital, Groby Road, Leicester LE3 9QP, UK

**Keywords:** Myocardial perfusion, Myocardial blood flow, Stress cardiovascular magnetic resonance, Left ventricular systolic dysfunction

## Abstract

**Aims:**

In patients with heart failure with reduced ejection fraction (HFrEF), determining the aetiology of cardiac dysfunction has important therapeutic and prognostic implications. Cardiovascular magnetic resonance (CMR) enables comprehensive phenotyping of HFrEF; however, it remains uncertain whether the choice of pharmacological stress agent influences the hyperaemic response required for reliable ischaemia assessment. We sought to compare the hyperaemic effects of adenosine and dobutamine in patients with HFrEF using quantitative perfusion CMR.

**Methods and results:**

Patients with HFrEF [left ventricular ejection fraction (LVEF) ≤40%] prospectively underwent 3-Tesla CMR comprising functional cine imaging, late gadolinium enhancement (LGE), and first-pass perfusion imaging at rest and during pharmacological stress with (i) adenosine (140–210 μg/kg/min) and (ii) dobutamine (10–30 µg/kg/min). Perfusion maps were reconstructed inline with automated, pixel-wise quantification of myocardial blood flow (MBF). The hyperaemic response, defined by global myocardial perfusion reserve (MPR), was calculated as the quotient of stress and rest MBF and compared between stress protocols. Fifty-three patients with HFrEF (mean age 63 ± 10 years, 77% male, mean LVEF 36 ± 10%, infarction 59%, and non-ischaemic focal fibrosis 21%) with paired adenosine and dobutamine stress–perfusion data were analysed. Compared with dobutamine, adenosine produced a higher global MPR [mean difference: +0.61 (95% CI: 0.35, 0.88); *P* < 0.001], which remained significant at the segmental level following adjustment for age, sex, type 2 diabetes, LVEF, and LGE presence [mean difference: +0.63 (95% CI: 0.55, 0.71); *P* < 0.001].

**Conclusion:**

In patients with HFrEF, adenosine induces a greater hyperaemic response than dobutamine; however, whether this impacts on the diagnostic assessment of ischaemia remains to be established (Trial registration: NCT03661827).

## Graphical Abstract

**Graphical Abstract:**
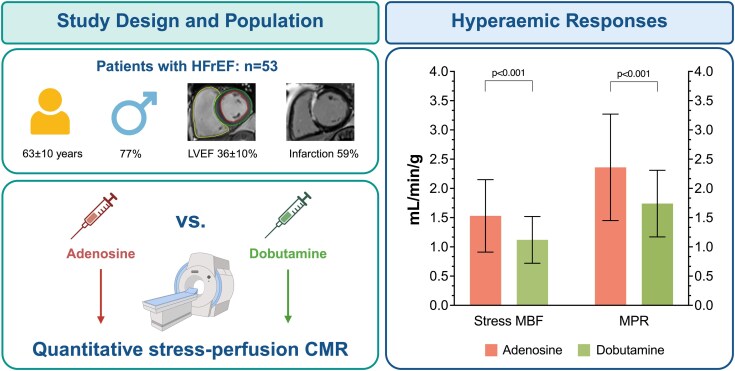

## Introduction

In patients with heart failure with reduced ejection fraction (HFrEF), determining the aetiology of cardiac dysfunction has important prognostic implications and may guide therapeutic interventions such as revascularization and/or device therapy.^1–3^ Cardiovascular magnetic resonance (CMR) enables comprehensive phenotyping of HFrEF, providing an integrated assessment of ventricular function, myocardial perfusion, and fibrosis within a single examination. While the diagnostic and prognostic value of stress–perfusion CMR is well established in patients with known or suspected coronary artery disease (CAD),^4^ its ability to detect significant CAD in patients with HFrEF remains uncertain.^5^

In clinical practice, adenosine is the most commonly used pharmacological vasodilator for stress CMR exams owing to its favourable safety profile and tolerability.^6,7^ However, in patients with left ventricular systolic dysfunction (LVSD), the haemodynamic and hyperaemic responses to adenosine may be attenuated,^8,9^ potentially compromising diagnostic sensitivity for the detection of myocardial ischaemia. Given the impaired vasodilatory reserve in HFrEF,^10,11^ dobutamine may provide a more effective means for inducing hyperaemia in this population. However, to date, no study has directly compared the hyperaemic effects of these two stress agents in patients with HFrEF.

Accordingly, in this prospective clinical study, we sought to compare the myocardial hyperaemic responses [defined as stress myocardial blood flow (MBF) and myocardial perfusion reserve (MPR)] to adenosine and dobutamine in patients with HFrEF using quantitative perfusion CMR, enabling an objective assessment of pharmacological stress responses beyond conventional haemodynamic surrogates.

## Methods

### Study design

Consecutive adult patients with HFrEF [left ventricular ejection fraction (LVEF) ≤ 40%] identified either by echocardiography or by CMR, who were awaiting or had undergone coronary angiographic assessment (within 6 months), were prospectively recruited. Participants were recruited from three centres across the UK, with all research investigations conducted at a single tertiary cardiac centre (Glenfield Hospital, Leicester, UK) between February 2019 and July 2023. A group of asymptomatic controls with LVEF >50% and CAD excluded on coronary computed tomography angiography were recruited for comparison. Exclusion criteria were recent myocardial infarction or unstable angina (≤3 weeks), contraindications to adenosine (second-/third-degree atrioventricular block, severe chronic obstructive pulmonary disease, moderate–severe asthma) or dobutamine (severe hypertension, uncontrolled arrhythmia, left ventricular outflow tract obstruction), severe renal dysfunction (estimated glomerular filtration rate <30 mL/min/1.73m^2^), severe claustrophobia, and absolute contraindications to CMR (non-MR-conditional cardiac implantable electronic device, pregnancy, ferromagnetic implants/foreign bodies). The study was approved by the UK National Research Ethics Service (REC reference 18/SC/0540) and registered on ClinicalTrials.gov (NCT03661827). The study was conducted in accordance with the Declaration of Helsinki, and all participants gave written informed consent prior to participation.

### Cardiovascular magnetic resonance

**Figure 1:**
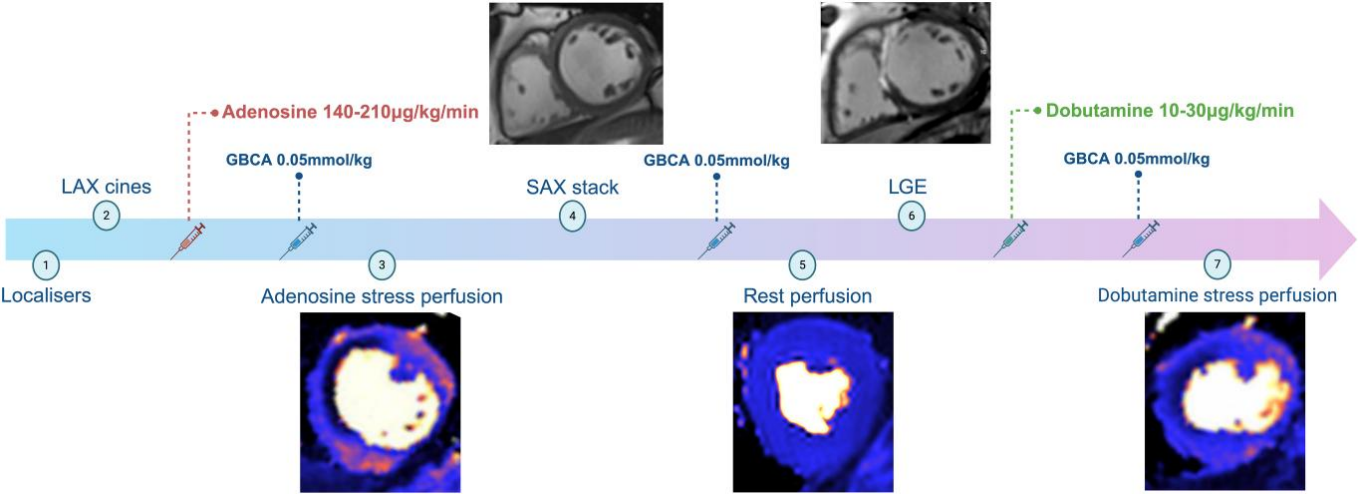
CMR protocol. *GBCA*, gadolinium-based contrast agent; *LAX*, long-axis; *LGE*, late gadolinium enhancement; *SAX*, short axis.

Participants underwent CMR at 3-Tesla (Vida or Skyra, Siemens Healthineers, Erlangen, Germany) with electrocardiographic gating and an 18-channel phased-array cardiac receiver coil (*Figure 1*). Participants were advised to abstain from caffeine-containing products for at least 24 h prior to pharmacological stress and rate-limiting medications (i.e. beta blockers and calcium channel blockers) for 48 h.

Following standard pilot and localizer imaging, functional cine imaging was performed in the three long-axis planes (four-, two-, three-chamber) and a contiguous short-axis stack to cover the ventricles using a breath-hold, steady-state free precession pulse sequence [typical sequence parameters: echo time (TE) 1.49 ms, repetition time (TR) 3.39 ms, temporal resolution 47.5 ms, slice thickness 8 mm, distance factor 25%, matrix 256 × 166, field of view (FOV) 360–400 mm, FOV phase 81.3%, flip angle 80°].

A dual-sequence T1-weighted saturation–recovery gradient echo sequence with fast low-angle shot readout for myocardial imaging was acquired over 60 heartbeats at rest and during pharmacological stress (typical sequence parameters: TE 1 ms, TR 2.1 ms, total imaging duration including saturation recovery and delay 146 ms per slice, slice thickness 8 mm, matrix 192 × 111, FOV 360–400 mm, FOV phase 75%, flip angle 14°) at three short-axis levels (basal, mid-ventricular, and apical), with injection of 0.05 mmol/kg gadoterate meglumine (Dotarem, Guerbet, France) for each perfusion scan (total dose 0.15 mmol/kg) at 4 mL/s, followed by a 20 mL 0.9% saline bolus.^12^ Inline automated reconstruction and image post-processing was implemented within the Gadgetron software framework, calculating MBF using a blood–tissue exchange model, displayed on pixel-wise perfusion maps.^13,14^

Late gadolinium enhancement (LGE) imaging was acquired using a single-shot, free-breathing, motion-corrected, steady-state free precession sequence (magnitude and phase-sensitive inversion recovery reconstructions) in the same long- and short-axis slice prescriptions as the cine imaging [typical sequence parameters: TE 1.16 ms, slice thickness 8 mm, distance factor 25%, matrix 240 × 144 (parallel imaging factor 2), FOV 360–420 mm, FOV phase 75%, flip angle 50°],^15^ with the optimal inversion time determined from a Look–Locker sequence.

### Pharmacological stress

During pharmacological stress, heart rate (HR), blood pressure, pulse oximetry, and symptoms were assessed and recorded at baseline and at 1-min intervals. Pharmacological stress was performed in a fixed order (adenosine followed by dobutamine), enabling both agents to be administered within a single imaging session. Hyperaemia was first induced with adenosine at a rate of 140 μg/kg/min for 3–5 min, with dose escalations at 2-min intervals to 170–210 μg/kg/min if there was an insufficient symptomatic and/or haemodynamic response [HR increase ≥10 beats per minute (bpm)].^16^ Following adenosine, there was a minimum target interval of 10 min before performing rest perfusion. Following LGE imaging, dobutamine was infused in progressive 5-min stages of 10–30 µg/kg/min (approximately 10 min after rest perfusion). If required, intravenous atropine sulphate (0.25–0.5 mg aliquots, up to a maximum total dose of 2 mg) was used at the intermediate stage (20 µg/kg/min) to augment the HR response if required (target HR 85% of the age-predicted maximum). After terminating the infusion, dobutamine was reversed with intravenous metoprolol.

### Image analysis

Cardiovascular magnetic resonance images were analysed offline, blinded to participant and case-control status using certified software (cvi42, v. 6.1.2; Circle Cardiovascular Imaging, Calgary, Canada). Volumetric analysis was performed with methods as previously described.^17^ LGE images were analysed by two experienced Level 3 CMR accredited observers acting in consensus using the 16-segment American Heart Association model.^18^ For qualitative LGE assessment, scar patterns were graded at a segmental level: 0 = normal, 1 = subendocardial infarction, 2 = transmural infarction, and 3 = non-ischaemic. For quantitative LGE assessment, infarction was quantified using the full width at half maximum technique and non-ischaemic focal fibrosis using the 5-standard deviation method.^19,20^

For quantitative perfusion analysis, inline, motion-corrected perfusion maps were analysed in a randomized order, blinded to qualitative perfusion, LGE, and clinical data. Raw and automatically segmented rest and stress (adenosine and dobutamine) pixel-wise perfusion maps were visually inspected. If there was significant artefact precluding analysis (e.g. outflow tract inclusion on the basal slice, wrap artefact or failure of motion correction), the affected segments were excluded. If the automated myocardial contours failed to appropriately detect the endocardial and epicardial contours, these were manually redrawn with a 10% offset applied to each border. MPR was calculated as the quotient of segmental stress and rest MBF, applying the 16-segment American Heart Association model, with segmental values averaged to derive a global value. All analysable segments, including those with infarction, were included in the global MBF and MPR calculations. As a single rest MBF acquisition was performed, this served as the denominator for both adenosine and dobutamine MPR. The hyperaemic response was defined as stress MBF and MPR, distinct from the peripheral haemodynamic response (HR, blood pressure, and rate–pressure product).

### Statistical analysis

Continuous data are expressed as mean ± standard deviation for normally distributed variables or as median [quartile (Q)1-Q3] for non-normally distributed variables. Normality was assessed using the Shapiro–Wilk test and with visual inspection of histograms and Q–Q plots. Categorical data are presented as counts and percentages (%). For the primary analysis, global MPR during adenosine and dobutamine stress was compared within HFrEF patients using paired-sample t-tests and mean differences [95% confidence interval (CI)], with effect sizes estimated using Cohen’s dz. Secondary analyses compared global stress MBF and haemodynamic responses. To account for the within-patient correlation of segmental perfusion data, linear mixed-effects models were used to assess segmental perfusion responses between stress methods, adjusting for key covariates. Random intercepts were specified for each patient and segment, with a variance components covariance structure used to account for the hierarchical clustering of segments within patients. A *P*-value <0.05 was considered statistically significant. Statistical analysis was performed using the Statistical Package for Social Sciences version 31.0 (IBM Corp. Armonk, NY, USA).

## Results

### Subject characteristics

**Figure 2:**
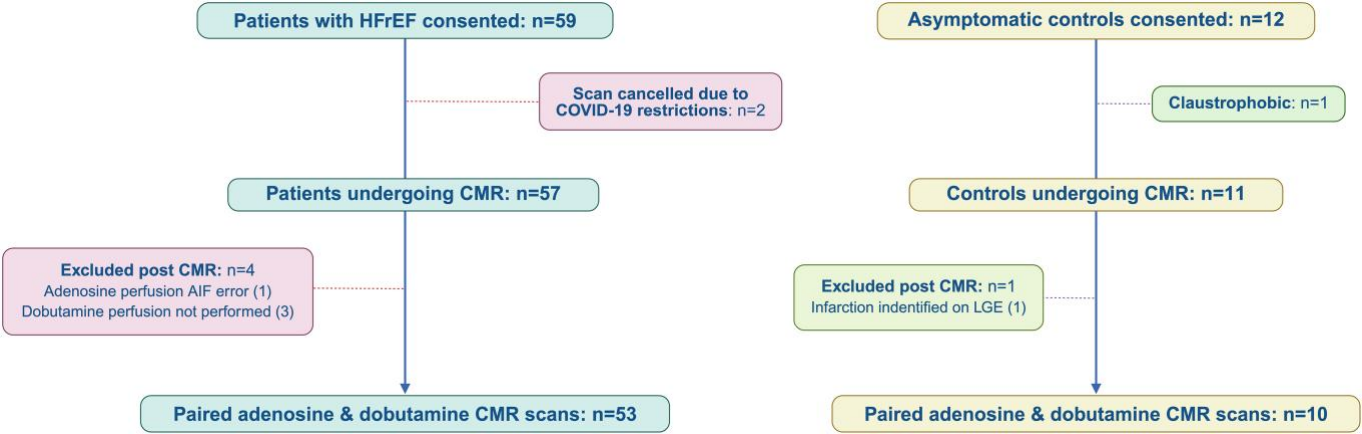
Study flow diagram. *AIF*, arterial input function; *CMR*, cardiovascular magnetic resonance; *HFrEF*, heart failure with reduced ejection fraction. ^†^Dobutamine perfusion not performed; residual left ventricular thrombus (1), recent acute myocardial infarction (1), and new diagnosis of hypertrophic obstructive cardiomyopathy (1).

Of 59 prospectively recruited patients with HFrEF, 53 were included in the final analysis (mean age 63 ± 10 years, 77% male, mean LVEF 36 ± 10%). Six patients were excluded: four did not have paired adenosine and dobutamine perfusion scans, and two had research visits cancelled due to the COVID-19 pandemic (*Figure 2*). Baseline characteristics of patients with HFrEF and control subjects are summarized in *Table 1*. When compared with controls (*n* = 10), HFrEF participants had a higher prevalence of cardiovascular risk factors and prescription medication use. In the HFrEF group, aetiology was predominantly ischaemic, with infarction present in 59% and non-ischaemic focal fibrosis in 21% (*Table 1*).

**Table 1:** Baseline characteristics

	HFrEF subjects (*n* = 53)	Control subjects (*n* = 10)
Demographics		
Age, years	63 ± 10	64 ± 7
Male	41 (77.4%)	5 (50.0%)
Body mass index, kg/m^2^	29.3 ± 5.3	26.7 ± 4.2
Cardiovascular risk factors		
Hypertension	27 (50.9%)	3 (30.0%)
Type 2 diabetes	13 (24.5%)	1 (10.0%)
Hypercholesterolaemia	18 (34.0%)	3 (30.0%)
Current or ex-smoker	34 (64.2%)	5 (50.0%)
Previous myocardial infarction	22 (41.5%)	0
Family history of premature CAD	26 (49.1%)	3 (30.0%)
Previous PCI	5 (9.4%)	0
Medications		
ACEi/ARB	45 (84.9%)	2 (20.0%)
Beta blocker	41 (77.4%)	2 (20.0%)
Calcium channel blocker	4 (7.5%)	2 (20.0%)
Diuretic	25 (47.2%)	0
Statin	32 (60.4%)	5 (50.0%)
Aspirin	24 (45.3%)	2 (20.0%)
P2Y_12_ inhibitor	19 (35.8%)	1 (10.0%)
Anti-coagulant	10 (18.9%)	0
Oral hypoglycaemic agent	13 (24.5%)	1 (10.0%)
Insulin	2 (3.8%)	0
Bloods		
Haemoglobin, g/L	143 ± 19	144 ± 13
Creatinine, mmol/L	84 [74–94]	81 [69–86]
NT-pro BNP, ng/L^a^	381 [119–1126]	48 [36–163]
Electrocardiogram		
Sinus rhythm	47 (88.7%)	10 (100%)
Atrial fibrillation	6 (11.3%)	0
QRS duration, ms	111 ± 26	99 ± 23
CMR		
*Left ventricular function*		
Ejection fraction, %	35.7 ± 10.1	58.8 ± 4.3
End-diastolic volume index, mL/m^2^	103.9 ± 25.1	75.4 ± 11.9
End-systolic volume index, mL/m^2^	68.2 ± 24.3	31.2 ± 6.2
Mass index, g/m^2^	65.9 ± 14.9	44.9 ± 7.3
*LGE*		
Infarction	31 (58.5%)	0
Enhanced mass, g	13.4 [6.9–25.1]	—
Non-ischaemic focal fibrosis	11 (20.8%)	0
Enhanced mass, g	8.8 [4.5–14.2]	—

Data presented as mean ± SD, median [Q1–Q3], or counts and percentages (%).

*ACEi/ARB*, angiotensin-converting enzyme inhibitor/angiotensin receptor blocker; *CAD*, coronary artery disease; *CMR*, cardiovascular magnetic resonance; *HFrEF*, heart failure with reduced ejection fraction; *LGE*, late gadolinium enhancement; *NT-pro BNP*, N-terminal pro-B-type natriuretic peptide; *PCI*, percutaneous coronary intervention.

^a^Lab reference range for NT-pro BNP: 20–200 ng/L

### Haemodynamic responses to pharmacological stress

When compared with controls, patients with HFrEF had similar haemodynamic responses with both adenosine (change in HR: 19 ± 10bpm in patients vs. 23 ± 9bpm in controls, *P* = 0.219) and dobutamine (47 ± 14bpm vs. 52 ± 27 bpm, respectively, *P* = 0.379) (*Table 2*). Within the HFrEF and control groups, baseline haemodynamic parameters were similar before administration of each pharmacological stress agent (*Table 3*, Supplementary data online, *Table S1* for control data). However, during peak stress, the HR and systolic blood pressure responses were greater with dobutamine than with adenosine (*Table 3*, Supplementary data online, *Table S1*).

**Table 2:** Haemodynamic effects of adenosine and dobutamine in HFrEF and control subjects

	HFrEF subjects (*n* = 53)	Control subjects (*n* = 10)	*P*-value
Adenosine^a^			
* Baseline*			
Heart rate, bpm	72 ± 13	62 ± 10	—
Systolic BP, mmHg	134 ± 22	137 ± 21	—
Diastolic BP, mmHg	81 ± 11	83 ± 8	—
*Peak stress*			
Heart rate, bpm	91 ± 15	85 ± 10	—
Systolic BP, mmHg	133 ± 23	132 ± 17	—
Diastolic BP, mmHg	79 ± 11	84 ± 33	—
Adenosine dose increase	19 (36%)	1 (9%)	—
*Δ Haemodynamics*			
Heart rate, bpm	19 ± 10	23 ± 9	0.219
Systolic BP, mmHg	−2 ± 18	−5 ± 15	0.663
Dobutamine^b^			
*Baseline*			
Heart rate, bpm	74 ± 14	68 ± 11	—
Systolic BP, mmHg	134 ± 23	135 ± 15	—
Diastolic BP, mmHg	79 ± 10	80 ± 7	—
*Peak stress*			
Heart rate, bpm	121 ± 15	120 ± 28	—
Systolic BP, mmHg	146 ± 35	148 ± 13	—
Diastolic BP, mmHg	78 ± 15	71 ± 9	—
*Δ Haemodynamics*			
Heart rate, bpm	47 ± 14	52 ± 27	0.379
Systolic BP, mmHg	12 ± 28	14 ± 19	0.853

*BP*, blood pressure; *BPM*, beats per minute; *HR*, heart rate.

^a^Baseline and peak adenosine BP unavailable for one HFrEF subject.

^b^Baseline and peak dobutamine haemodynamics unavailable for one HFrEF subject and baseline and peak BP not available for one control subject.

**Table 3:** Comparison of the haemodynamic and hyperaemic response to adenosine and dobutamine in HFrEF subjects

Haemodynamic response	Adenosine		Dobutamine		Mean difference [95% CI]
Baseline					
Heart rate, bpm	73 ± 13		74 ± 14		−1 [−3, 1]
Systolic BP, mmHg	135 ± 21		134 ± 22		1 [−4, 6]
Diastolic BP, mmHg	82 ± 11		79 ± 10		2 [−1, 5]
Rate–pressure product	9692 ± 2056		9745 ± 1947		−53 [−511, 405]
Peak stress					
Heart rate, bpm	91 ± 15		121 ± 15		−29 [−33, −26]
Systolic BP, mmHg	134 ± 22		146 ± 35		−12 [−20, −4]
Diastolic BP, mmHg	79 ± 11		78 ± 15		1 [−4, 6]
Rate–pressure product	12 199 ± 2740		17 677 ± 4884		−5478 [−6547, −4409]

Hyperaemic response	Adenosine	Dobutamine	*P*-value	Mean difference	Cohen’s dz
Global					
Stress MBF, mL/min/g	1.53 ± 0.62	1.12 ± 0.40	<0.001	0.40 [0.24, 0.57]	0.660
MPR	2.36 ± 0.91	1.74 ± 0.57	<0.001	0.61 [0.35, 0.88]	0.642

BP, blood pressure; *BPM*, beats per minute; *HR*, heart rate; *MBF*, myocardial blood flow; *MPR*, myocardial perfusion reserve.

### Myocardial hyperaemic responses to pharmacological stress

In patients with HFrEF, overall image quality of the perfusion maps was high, with four segments excluded at rest (0.5%), seven (0.8%) during adenosine stress, and 13 (1.5%) during dobutamine stress. One complete adenosine stress dataset required manual recontouring. There was no significant difference in rest MBF between HFrEF and control subjects (0.66 ± 0.14 mL/min/g vs. 0.65 ± 0.12 mL/min/g, respectively, *P* = 0.826) (see Supplementary data online, *Table S2*).

With adenosine stress, the hyperaemic response in HFrEF was similar to that in control subjects, with comparable stress MBF (1.53 ± 0.62 mL/min/g vs. 1.69 ± 0.28 mL/min/g, respectively, *P* = 0.193) and MPR (2.36 ± 0.91 vs. 2.66 ± 0.36, respectively, *P* = 0.088) (see Supplementary data online, *Table S2*). However, with dobutamine stress, the hyperaemic response was attenuated in HFrEF compared with control subjects, for both stress MBF (1.12 ± 0.40 mL/min/g vs. 1.98 ± 1.02 mL/min/g, respectively, *P* = 0.026) and MPR (1.74 ± 0.57 vs. 3.07 ± 1.37, respectively, *P* = 0.014) (see Supplementary data online, *Table S2*).

**Figure 3:**
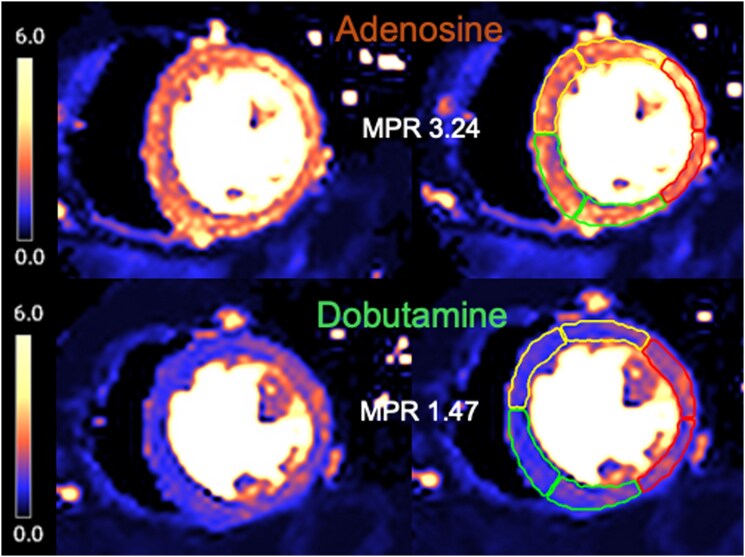
Representative example of paired adenosine and dobutamine unsegmented and segmented stress–perfusion maps at the basal ventricular level. A 64-year-old female with HFrEF (LVEF 32%) demonstrated a significant haemodynamic response to both adenosine (HR increase 27bpm) and dobutamine (HR increase 35bpm). However, the hyperaemic response was lower with dobutamine (MPR 1.47) compared with adenosine (MPR 3.24). *HFrEF*, heart failure with reduced ejection fraction; *HR*, heart rate; *LVEF*, left ventricular ejection fraction; *MPR*, myocardial perfusion reserve.

**Figure 4:**
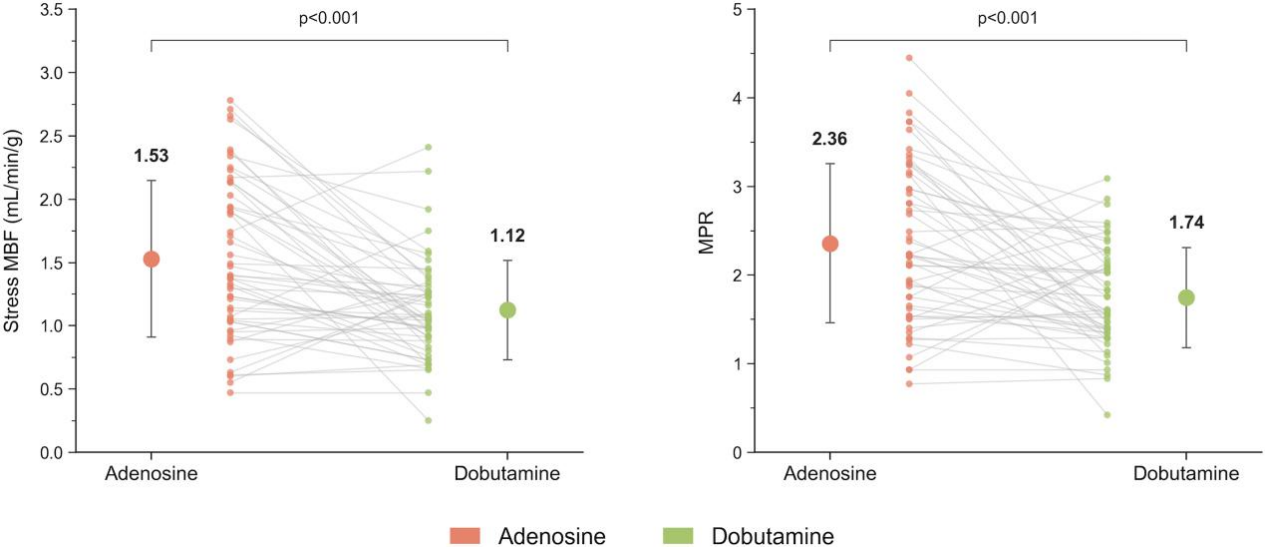
Paired comparisons of stress MBF and MPR during adenosine and dobutamine stress in patients with HFrEF. *HFrEF*, heart failure with reduced ejection fraction; *MBF*, myocardial blood flow; *MPR*, myocardial perfusion reserve.

When comparing the hyperaemic responses within HFrEF patients, compared with dobutamine, adenosine produced a greater increase in global stress MBF [mean difference: +0.40 mL/min/g (95% CI: 0.24, 0.57), *P* < 0.001] and MPR [mean difference: +0.61 (95% CI: 0.35, 0.88), *P* < 0.001] (*Table 3*, *Figures 3* and *4*), with comparable differences observed irrespective of the presence of infarction, although attenuated in those with prior infarction (see Supplementary data online, *Table S3*).

In contrast, the hyperaemic responses were similar for both stressors in control participants, with no significant difference in global stress MBF [mean difference: −0.29 mL/min/g (95% CI: −1.00, 0.41), *P* = 0.467] and MPR [mean difference: −0.41 (95% CI: −1.41, 0.59), *P* = 0.889] (see Supplementary data online, *Table S1*).

### Segmental myocardial perfusion responses to pharmacological stress

In a segmental level, linear mixed-effects model adjusted for age, sex, type 2 diabetes, LVEF, infarction, and non-ischaemic focal fibrosis, adenosine produced a greater hyperaemic response compared with dobutamine in participants with HFrEF [stress MBF mean difference: +0.42 mL/min/g (95% CI: 0.37, 0.46), *P* < 0.001; MPR mean difference: +0.63 (95% CI: 0.55, 0.71); *P* < 0.001] (*Table 4*).

**Table 4:** Adjusted associations between clinical variables and segmental perfusion data based on a linear mixed-effects model in HFrEF subjects

Parameter	Estimate (β)*	95% CI	*P*-value
***MPR***			
Method (adenosine vs. dobutamine)	0.630	[0.549, 0.712]	<0.001
Age	−0.009	[−0.014, −0.004]	<0.001
Sex (males vs. females)	0.235	[0.108, 0.362]	<0.001
Type 2 diabetes	−0.254	[−0.367, −0.141]	<0.001
LVEF	0.006	[0.001, 0.011]	0.023
Infarction	−0.375	[−0.479, −0.270]	<0.001
Non-ischaemic focal fibrosis	−0.155	[−0.283, −0.027]	0.018
***Stress MBF***			
Method (adenosine vs. dobutamine)	0.415	[0.366, 0.464]	<0.001
Age	−0.005	[−0.008, −0.002]	0.003
Sex (males vs. females)	−0.045	[−0.129, 0.039]	0.291
Type 2 diabetes	−0.189	[−0.264, −0.114]	<0.001
LVEF	0.008	[0.005, 0.012]	<0.001
Infarction	−0.269	[−0.338, −0.200]	<0.001
Non-ischaemic focal fibrosis	−0.014	[−0.099, 0.071]	0.746

*β-coefficients represent adjusted mean differences for categorical variables and a one unit increase in continuous variables.

LVEF, left ventricular ejection fraction; MBF, myocardial blood flow; MPR, myocardial perfusion reserve.

## Discussion

In this prospective clinical study, we demonstrate that in patients with HFrEF, adenosine elicits a greater hyperaemic response compared with dobutamine. To our knowledge, this represents the first comparative study to evaluate these two pharmacological stress agents using quantitative perfusion CMR, providing an objective assessment of hyperaemia beyond peripheral haemodynamic surrogates. These findings provide important physiological insight, indicating that despite an impaired vasodilatory reserve in HFrEF, adenosine elicits a greater hyperaemic response than dobutamine. However, whether this translate into clinically meaningful differences in the detection of CAD in this population remains to be determined.

In HFrEF, the choice of pharmacological stress agent for the non-invasive assessment of ischaemia remains challenging. Peripheral haemodynamic responses are recommended to gauge hyperaemia,^16^ yet they are known to be unreliable.^21–23^ Consistent with this, dobutamine elicited a greater haemodynamic response, whereas adenosine induced a greater hyperaemic response. This discordance between peripheral haemodynamic and myocardial perfusion responses reinforces the limitations of relying on clinical haemodynamic markers to infer the adequacy of coronary vasodilation. These findings provide important context for our previous work, which demonstrated that patients with severe LVSD (LVEF ≤35%, *n* = 251) demonstrate a blunted haemodynamic response to adenosine,^8^ and in a separate cohort of 291 patients, LVEF independently predicts the likelihood of achieving a satisfactory hyperaemic response.^9^ Together, these observations highlight the uncertainty regarding the optimal pharmacological stress agent in patients with HFrEF.

Despite the absence of previous quantitative perfusion CMR studies directly comparing these two stressors, prior work has examined the hyperaemic response to adenosine in patients with HFrEF and to dobutamine in patients with CAD. Firstly, in a single-centre study of 24 patients with HFrEF (LVEF <40%), the effects of standard- (140 μg/kg/min) and high-dose (210 μg/kg/min) adenosine infusions on quantitative myocardial perfusion were compared.^24^ Despite similar haemodynamic responses between infusion protocols, high-dose produced a greater hyperaemic response than standard-dose adenosine (MPR: 2.26 ± 0.90 vs. 1.90 ± 0.88, respectively, *P* = 0.004). Similarly, a CMR study of 23 patients with angiographically proven significant CAD found that dobutamine provoked significant differences in stress MBF between ischaemic and remote segments in patients with single-vessel disease (0.90 ± 0.18 mL/min/g vs. 1.73 ± 0.32 mL/min/g, respectively, *P* < 0.001), and when compared with controls (*n* = 4), ischaemic segments demonstrated a blunted hyperaemic response (0.97 ± 0.20 mL/min/g vs. 2.0 ± 0.39 mL/min/g in controls, *P* < 0.001).^25^ Thus, while adenosine can elicit a measurable hyperaemic response in HFrEF, and dobutamine-induced myocardial perfusion can differentiate ischaemic from non-ischaemic segments, no direct evidence exists comparing the hyperaemic responses of these two approaches using contemporary CMR techniques.

However, a previous positron emission tomography (PET) study, although not in patients with HFrEF, demonstrated that in 13 patients with CAD, adenosine produced a higher peak absolute hyperaemic MBF than dobutamine in normal segments (3.10 ± 0.90 vs. 2.16 ± 0.99 mL/min/g, respectively, *P* < 0.001), with a similar pattern observed in ischaemic segments.^26^ Furthermore, in another PET study of 36 patients with CAD and 18 healthy volunteers, although not a within-patient comparison, stress MBF and MPR were significantly higher with adenosine than with dobutamine in controls and remote myocardium, while these differences were similarly attenuated in ischaemic territories.^27^ However, these findings are limited by the unpaired assessment of the stress agents, small sample size, and the absence of patients with HFrEF. By contrast, a study of 47 patients with suspected or known CAD with coronary flow velocity reserve as assessed by transthoracic echocardiography under both adenosine and dobutamine stress reported similar responses with the two agents (2.5 ± 0.7 vs. 2.4 ± 0.7, respectively, *P* = 0.70).^28^

Adenosine induces potent coronary vasodilation through activation of A_2_A receptors on vascular smooth muscle, resulting in an up to four-fold increase in MBF, facilitating the detection of regional flow heterogeneities attributable to micro- or macro-vascular CAD.^29^ Dobutamine is a widely used alternative pharmacological stress agent for the assessment of CAD, primarily by inducing ischaemic wall–motion abnormalities or to predict myocardial functional recovery following revascularization.^30,31^ As a synthetic catecholamine, dobutamine increases cardiac workload predominantly through β-adrenergic receptor stimulation, producing positive inotropic and chronotropic effects, with additional direct and indirect vasodilatory effects that increase myocardial oxygen demand and drive a demand-mediated increase in MBF, provoking regional perfusion defects in the presence of flow-limiting CAD.^25,32^

In HFrEF, downregulation of adenosine receptor gene expression, impaired adenosine signalling, and altered endothelium-dependent vasodilation have been suggested to attenuate the response to adenosine.^10,11^ Similarly, chronic sympathetic activation in HFrEF may result in β-adrenergic receptor desensitization, diminishing contractile response, which may also limit the ability of dobutamine to augment MBF.^33,34^ Although limited by the small control cohort, the hyperaemic response to dobutamine appeared blunted in HFrEF compared with adenosine, whereas responses were similar between stressors in controls. Notably, rest MBF was similar between HFrEF and control subjects, indicating that the attenuated hyperaemic response to dobutamine in HFrEF was not a consequence of lower baseline perfusion. In HFrEF, the sympathetic stimulation from dobutamine may exert several haemodynamic effects that adversely affect myocardial perfusion. Compared with healthy controls, patients with HFrEF have remodelled ventricles with impaired compliance, a higher burden of diffuse and focal fibrosis, and coexistent pulmonary vascular and microvascular disease. Dobutamine-mediated sympathetic stimulation may further worsen left ventricular filling pressures and increase afterload—collectively, these factors may contribute to an attenuated hyperaemic response and impair coronary microvascular vasodilatory reserve, potentially limiting the detection of ischaemia. This may in part explain the differing hyperaemic responses to dobutamine observed between HFrEF patients and control subjects.

Despite the clear mechanistic differences between these stressors, and the potential for impaired coronary hyperaemia with these agents in HFrEF, our data demonstrate that adenosine produces a greater hyperaemic response than dobutamine. This suggests that adenosine may be a more reliable stress agent for myocardial perfusion assessment in this population; however, whether the relationship between the magnitude of hyperaemia translates into the improved detection of ischaemia remains to be established.

## Study limitations

Despite the prospective nature with blinded analysis, this study has several limitations. Firstly, the sample size was relatively modest, and due to the lack of robust field-, vendor-, and model-specific myocardial perfusion data and established normative reference ranges, we were unable to utilize an *a priori* definition of equivalence to power our findings. Secondly, although participants were instructed to abstain from caffeine and negative inotropic medications prior to CMR, adherence was not strictly controlled and relied on participant compliance. This may have influenced the hyperaemic response to pharmacological stress, with previous studies showing that up to 20% of patients have detectable caffeine levels despite reported abstention.^35^ Our cohort predominantly included patients with moderate–severe LVSD, and in contrast to prior work, the haemodynamic response of HFrEF patients was not blunted in patients compared with controls. Consequently, the hyperaemic response may be further attenuated in patients with more advanced heart failure, highlighting the need for further investigation in populations with severe LVSD. We did not assess splenic switch-off as an alternative marker of adenosine stress adequacy; however, it has been recognized that both HR and splenic switch-off are imperfect markers of hyperaemia.^23^ Additionally, the inclusion of patients who had undergone or were awaiting angiographic assessment may introduce selection bias towards patients with suspected CAD, limiting generalizability towards broader HFrEF populations. Furthermore, the sequence of adenosine and dobutamine administration was not randomized, as the protocol was prespecified to perform both studies within a single imaging session to minimize interstudy variability in perfusion that may occur with imaging on different days and for logistical reasons in light of the pandemic. However, a sufficient time interval followed adenosine infusion to ensure adequate washout, and any residual hyperaemia would be expected to be reflected in the single rest MBF and accounted for in the calculation of MPR for both stress agents. Finally, while this study provides mechanistic insight into the relative performance of these two stressors in patients with HFrEF, it was neither designed, nor was it intended to evaluate diagnostic performance.

## Conclusions

In patients with HFrEF, compared with dobutamine, adenosine produces a greater hyperaemic response, supporting its effectiveness as a stress agent in this population. However, the clinical impact of these differences on myocardial ischaemia detection remains to be established. Further prospective studies evaluating the diagnostic performance of adenosine and dobutamine stress–perfusion CMR against invasive reference standards are warranted.

## Supplementary Material

qyag128_Supplementary_Data

## Data Availability

The data underlying this article will be shared on reasonable request to the corresponding author.

## Lead author biography

Dr Simran Shergill graduated from the University of Birmingham in 2015. He completed his MRCP in 2018 and entered Cardiology specialty training in 2021. He is currently undertaking a period of research at the University of Leicester and NIHR Biomedical Research Centre: Leicester. His research focus involves the application of perfusion imaging and novel CMR techniques in patients with stable CAD.
