# Supplementary material for: Hyperaemic effects of adenosine and dobutamine in heart failure with reduced ejection fraction: a quantitative perfusion CMR study
Source: Eur Heart J Imaging Methods Pract. 2026 Jul 16;4(3):qyag128. doi: 10.1093/ehjimp/qyag128 (PMC13431899; doi:10.1093/ehjimp/qyag128)
Supplement: qyag128_Supplementary_Data [file qyag128_supplementary_data.docx]

| **Haemodynamic response** | **Adenosine** | **Dobutamine** | **Mean difference [95% CI]** | | |
| --- | --- | --- | --- | --- | --- |
| **Baseline** |  |  |  | | |
| Heart rate, bpm | 62 ± 10 | 68 ± 11 | -6 [-13, 1] | | |
| Systolic BP, mmHg | 134 ± 21 | 135 ± 15 | 0 [-7, 6] | | |
| Diastolic BP, mmHg | 82 ± 9 | 80 ± 7 | 2 [-3, 7] | | |
| Rate-pressure product | 8277 ± 2029 | 9240 ± 1732 | -963 [-1789, -137] | | |
| **Peak stress** |  |  |  | | |
| Heart rate, bpm | 85 ± 10 | 120 ± 28 | -36 [-54, -17] | | |
| Systolic BP, mmHg | 130 ± 16 | 148 ± 13 | -18 [-36, 1] | | |
| Diastolic BP, mmHg | 85 ± 35 | 71 ± 9 | 14 [-15, 43] | | |
| Rate-pressure product | 11239 ± 2033 | 18595 ± 4762 | -7356 [-11540, -3172] | | |
| **Hyperaemic response** | **Adenosine** | **Dobutamine** | **Mean difference** | **P value** | **Cohen’s dz** |
| **Global** |  |  |  |  |  |
| Stress MBF, mL/min/g | 1.69 ± 0.28 | 1.98 ± 1.02 | -0.29 [-1.00, 0.41] | 0.467 | -0.297 |
| MPR | 2.66 ± 0.36 | 3.07 ± 1.37 | -0.41 [-1.41, 0.59] | 0.889 | -0.295 |

**Supplementary Table 1**: Comparison of the haemodynamic and hyperaemic response to adenosine and dobutamine in control subjects

**Abbreviations:** *BP* blood pressure*; BPM* beats per minute; *HR* heart rate; *MBF* myocardial blood flow; *MPR* myocardial perfusion reserve.

**Supplementary Table 2:** Comparison of the global hyperaemic response to adenosine and dobutamine between HFrEF and control subjects

| **Hyperaemic response** | **HFrEF, n=53** | **Controls, n=10** | **Mean difference** | **P value** |
| --- | --- | --- | --- | --- |
| Rest MBF, mL/min/g | 0.66 ± 0.14 | 0.65 ± 0.12 | 0.01 [-0.08, 0.11] | 0.826 |
| ***Adenosine*** |  |  |  |  |
| Stress MBF, mL/min/g | 1.53 ± 0.62 | 1.69 ± 0.28 | -0.16 [-0.41, 0.09] | 0.193 |
| MPR | 2.36 ± 0.91 | 2.66 ± 0.36 | -0.30 [-0.64, 0.05] | 0.088 |
| ***Dobutamine*** |  |  |  |  |
| Stress MBF, mL/min/g | 1.12 ± 0.40 | 1.98 ± 1.02 | -0.86 [-1.60, -0.13] | 0.026 |
| MPR | 1.74 ± 0.57 | 3.07 ± 1.37 | -1.32 [-2.31, -0.34] | 0.014 |

**Abbreviations:** *HFrEF* heart failure with reduced ejection fraction*; MBF* myocardial blood flow; *MPR* myocardial perfusion reserve.

**Supplementary Table 3:** Comparison of the hyperaemic response to adenosine and dobutamine stratified by presence of infarction in HFrEF subjects

| **Hyperaemic response** | **Adenosine** | **Dobutamine** | **Mean difference** | **P value** | **Cohen’s dz** |
| --- | --- | --- | --- | --- | --- |
| **No infarction, n=22** |  |  |  |  |  |
| ***Global*** |  |  |  |  |  |
| Stress MBF, mL/min/g | 1.84 ± 0.69 | 1.19 ± 0.30 | 0.65 [0.36, 0.94] | <0.001 | 0.987 |
| MPR | 2.72 ± 0.95 | 1.79 ± 0.44 | 0.93 [0.49, 1.37] | <0.001 | 0.939 |
| **Infarction, n=31** |  |  |  |  |  |
| ***Global*** |  |  |  |  |  |
| Stress MBF, mL/min/g | 1.31 ± 0.48 | 1.07 ± 0.45 | 0.23 [0.04, 0.43] | 0.020 | 0.442 |
| MPR | 2.10 ± 0.79 | 1.71 ± 0.66 | 0.39 [0.07, 0.71] | 0.020 | 0.442 |
